# Supplementary material for: COVID-19 outbreaks in nursing homes: A strong link with the coronavirus spread in the surrounding population, France, March to July 2020
Source: PLoS One. 2022 Jan 7;17(1):e0261756. doi: 10.1371/journal.pone.0261756 (PMC8741027; doi:10.1371/journal.pone.0261756)

**S1 Fig.** Observed and modelled cumulative curves of COVID-19 outbreaks in the nursing homes of each *Département* of Auvergne-Rhône-Alpes Region over the study period (March 1 – July 31, 2020)

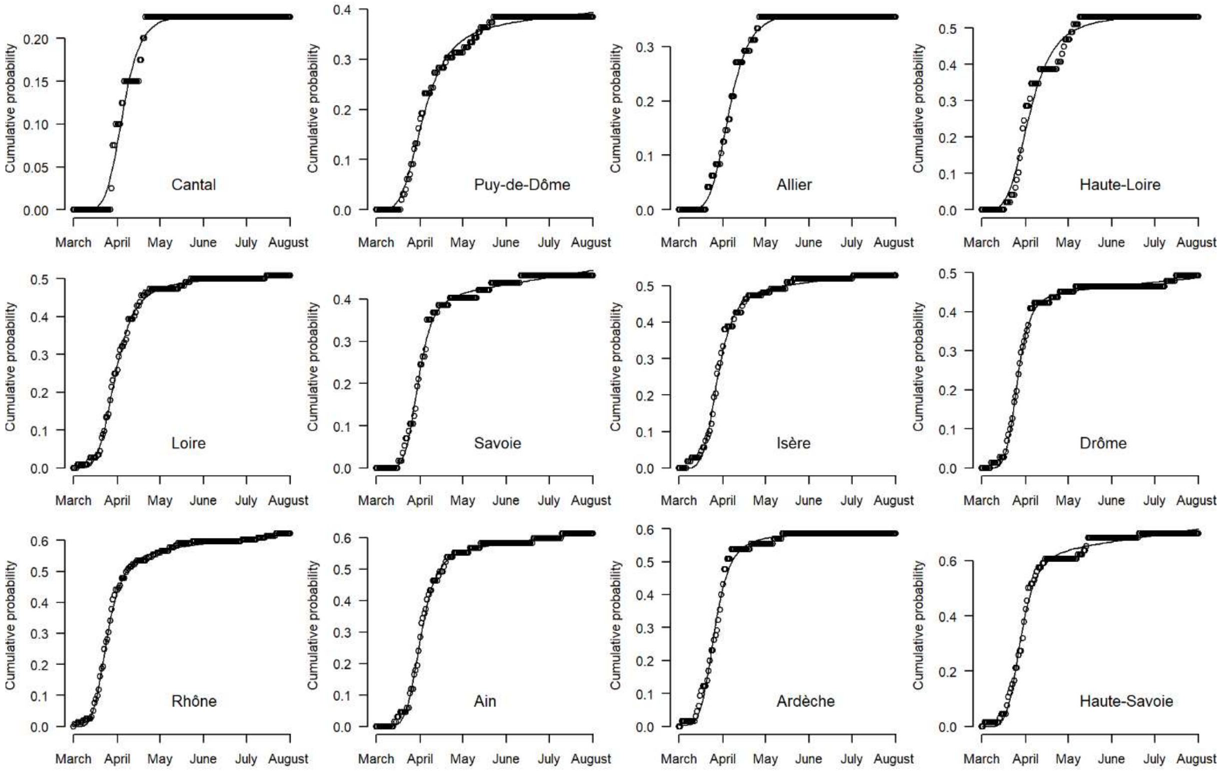

Supplement: S1 Fig — (PDF) [file pone.0261756.s001.pdf]
