# Supplementary material for: COVID-19 outbreaks in nursing homes: A strong link with the coronavirus spread in the surrounding population, France, March to July 2020
Source: PLoS One. 2022 Jan 7;17(1):e0261756. doi: 10.1371/journal.pone.0261756 (PMC8741027; doi:10.1371/journal.pone.0261756)

S3 Fig. Weekly dynamics of COVID-19 outbreaks in the nursing homes of Auvergne-Rhône-Alpes Region over the study period (943 nursing homes; March 1 – July 31, 2020). The percentages correspond to the changes in the proportion of nursing homes with outbreaks

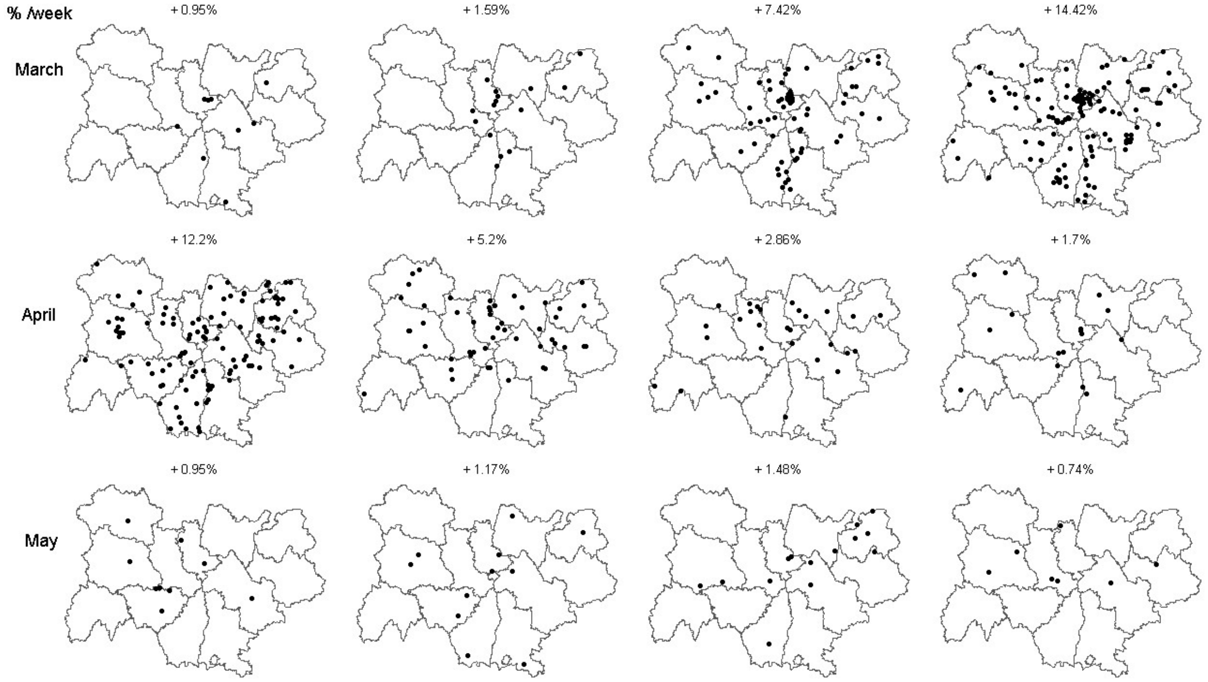

Supplement: S3 Fig — (PDF) [file pone.0261756.s003.pdf]
